# Altered Hippocampal Transcriptomic Profile Reveals Cognitive Impairment in Young Metabolically Obese, Normal‐Weight Rats, Prevented by Perinatal Leptin Intake

**DOI:** 10.1002/mnfr.70262

**Published:** 2025-09-13

**Authors:** Carmen García‐Ruano, Andrea Costa, Andreu Palou, Paula Oliver

**Affiliations:** ^1^ Nutrigenomics Biomarkers and Risk Evaluation (NuBE) Group University of the Balearic Islands (UIB), Palma Mallorca Spain; ^2^ Health Research Institute of the Balearic Islands (IdISBa), Palma Mallorca Spain; ^3^ CIBER of Physiopathology of Obesity and Nutrition (CIBEROBN) Instituto De Salud Carlos III Madrid Spain; ^4^ Eurecat, Centre Tecnològic de Catalunya Technological Unit of Nutrition and Health Reus Spain

**Keywords:** cognition, early biomarkers, obesity phenotypes, PBMC, perinatal programming

## Abstract

Early‐life metabolic environment significantly impacts long‐term cognitive and metabolic health. This study investigates transcriptomic alterations in the hippocampus and peripheral blood mononuclear cells (PBMC) of young rats exposed to an isocaloric high‐fat diet (HFD), resulting in the metabolically obese, normal‐weight (MONW) phenotype.

Rats were pair‐fed either a standard (NW group) or HFD (MONW group) for 11 weeks after weaning. Another group (MONW‐Lep) received leptin supplementation during lactation and subsequently HFD. Transcriptomic analysis of the hippocampus showed disruption of pathways linked to obesity and cognitive decline in the MONW group, which were attenuated by leptin intake. This was consistent with the results of working memory (T‐maze test), impaired in MONW versus NW, but preserved in MONW‐Lep animals. PBMC transcriptomics revealed overlapping genes with the hippocampus. Notably, *Piwil1*, a gene linked to neurodegeneration, metabolic syndrome and obesity, was up‐regulated in PBMC of MONW but not of MONW‐Lep animals, reflecting early hippocampal changes and leptin's preventive effect. These findings highlight the influence of early nutrition on cognitive health, the protective potential of leptin counteracting the effects of HFD intake and the usefulness of PBMC as a reliable source of biomarkers of brain health.

## Graphical abstract

Early‐life exposure of rats to high‐fat‐isocaloric diet leads to metabolically obese, normal‐weight phenotype, and impaired cognition. Hippocampal transcriptomic analysis reveals disruption in pathways linked to obesity and cognitive decline, which is mitigated by perinatal leptin supplementation. PBMC share common altered genes with hippocampus, which are potentially useful as minimally‐invasive biomarkers for cognitive risk. Notably, *Piwil1*, linked to obesity and cognitive decline, mirrors hippocampal changes in PBMC at an early stage.

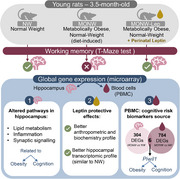

**Abbreviations**
AD: Alzheimer's disease
DEGs: differentially expressed genes
HFD: high‐fat diet
MONW: metabolically obese, normal‐weight
NW: normal weight
PBMC: peripheral blood mononuclear cells

## 1 Introduction

The relationship between early‐life nutrition and long‐term health outcomes has gained increasing attention in recent years. Poor dietary habits during critical developmental periods are known to influence not only metabolic health [1] but also cognitive function [2]. In this line, early exposure to an unbalanced macronutrient diet, such as a high‐fat diet (HFD), even under isocaloric conditions, can lead to the development of the metabolically obese, normal‐weight (MONW) phenotype [3]. This condition is characterized by increased visceral adiposity, insulin resistance, and hepatic lipid accumulation in the absence of overt obesity [4]. Beyond these metabolic features, our group was pioneer to demonstrate, in rodents, that the MONW phenotype is linked to impairments in cognitive functions, particularly those involving the hippocampus, a brain region critical for memory and learning [5].

The hippocampus's sensitivity to metabolic changes makes it a key area for studying the cognitive impacts of early‐life dietary challenges [2]. In fact, we previously demonstrated that not only adult but also young rats exposed to an isocaloric HFD exhibited MONW characteristics and cognitive deficits [6]. These impairments were evident in tasks requiring working memory and were reflected at the transcriptional level in peripheral blood mononuclear cells (PBMC) [6]. PBMC have proven to be a reliable source of biomarkers for metabolic disease and cognitive decline [6, 7]. Additionally, PBMC have been shown to reflect altered gene expression patterns occurring in the hippocampus of patients with Alzheimer's disease (AD) [8]. However, the specific molecular mechanisms within the hippocampus that link early metabolic stress to cognitive dysfunction remain unclear.

One potential intervention to counteract these adverse effects caused by early malnutrition consists in the administration of leptin, a hormone present in maternal milk that plays a crucial role in perinatal programming [9]. Perinatal leptin intake has been shown to protect against obesity (reviewed in [10]), and against the MONW phenotype in adult rats [11]. Leptin not only regulates energy balance but also plays a critical role in hippocampus development, where it acts as a cognitive enhancer [12]. Leptin receptors are widely expressed in the hippocampus, specifically, within this brain region, the functional long isoform has been identified in pyramidal neurons, astrocytes, microglia, and also in neural progenitor cells, supporting the role of leptin in modulating synaptic plasticity, promoting adult neurogenesis and contributing to improved memory performance [13, 14, 15, 16, 17]. Interestingly, the long isoform of leptin receptor is also expressed in PBMC, including both lymphocytes and monocytes, enabling these cells to respond to the hormone [18, 19]. In our earlier studies, neonatal leptin supplementation during lactation demonstrated protective effects against cognitive and metabolic impairments induced by early HFD exposure, with alterations at the hippocampal level that were somehow reflected by PBMC [6]. While our prior work focused on the analysis of specific genes of interest [6], in the current study we perform a whole‐genome transcriptomic analysis in the hippocampus, aiming to uncover the molecular pathways affected by early HFD intake linked to MONW phenotype and to assess the potential mitigating effects of leptin. By linking the cognitive consequences of early HFD exposure to specific molecular signatures, our study provides deeper insights into how prompt metabolic challenges affect brain health. Additionally, this research assesses whether leptin supplementation can mitigate these alterations and explores the potential of PBMC as an accessible source of biomarkers that reflect brain changes. Based on our previous findings, we hypothesize that early HFD intake induces transcriptomic changes associated with cognitive impairment, that these alterations are prevented by perinatal leptin intake, and that such changes can be detected both in the hippocampus and PBMC.

## 2 Experimental Section

### 2.1 Animal and Experimental Design

The animal study protocol was conducted in accordance with the Declaration of Helsinki and approved by the Bioethical Committee of the University of the Balearic Islands (Exp.: 2015/23/AEXP, approved December 28, 2015). University guidelines for the use and care of laboratory animals were followed. Animals were purchased from Charles River Laboratories (Barcelona, Spain). The experimental design has been previously described in detail [11]. Briefly, male Wistar rats received either vehicle (*n* = 16) or oral leptin supplementation (*n* = 10) during lactation (postnatal days 1–20). The amount of leptin administered to the animals was calculated as five times the average amount of daily leptin intake from maternal milk, calculated in a previous study by our group [20]. After weaning, rats who received the vehicle were pair‐fed under isocaloric conditions with either a control normolipidic diet with 10% calories from fat (NW group, *n* = 8) or a HFD, with 60% calories from fat (MONW group, *n* = 8) for 11 weeks. Leptin‐treated animals were also fed the HFD under isocaloric conditions (MONW‐Lep group, *n* = 10). Diets were purchased from Brogaarden (Gentofte, Denmark); the complete diet composition (macronutrient proportion and ingredients) and fatty acid profile are detailed in [21].

The sample size was selected based on similar experimental designs, which show that this size has sufficient statistical power to detect statistical differences between groups [5, 21]. At 14 weeks (i.e., 3.5 months) animals were killed by decapitation within the first 2 h of the light cycle (8.00–10.00 h) in a fed (ad libitum) state. The hippocampus, liver, and various adipose tissues were collected. Moreover, PBMC were obtained as described below at 2 and 3.5 months of age. This animal design is part of a larger study on the impact of young MONW phenotype and neonatal treatments on metabolic health and cognition [6, 11]; however, hippocampus transcriptome analysis is novel to this work.

### 2.2 Behavioral Testing: T‐maze Alternation

The effect of the MONW phenotype and the leptin‐treatment on cognitive function was assessed using a spontaneous alternation paradigm in a T‐maze, as we described before [6]. The protocol from Deacon and Rawlins [22] was followed. Briefly, spontaneous alternation is a short‐term memory task that assesses an animal's ability to remember the arm in a T‐maze that the animal has previously entered and to select an alternative maze arm when re‐exposed to the device.

### 2.3 Adiposity Parameters

Body composition was measured using an EchoMRI‐700 (Echo Medical Systems, LLC, TX, USA) without anesthesia. Fat mass was measured and expressed as a percentage of total body weight. Visceral fat content was calculated as the sum of epididymal, mesenteric, and retroperitoneal white adipose tissue depot weights, and also expressed as a percentage of total body weight. In addition, lipid extracts from the liver were used to calculate total liver fat content, as previously described [11].

### 2.4 Blood Collection and PBMC Isolation

Blood samples were collected in the fed state at 2 and 3.5 months for PBMC isolation using Optiprep (Sigma Aldrich Química SA, Madrid, Spain) density gradient separation according to the manufacturer's instructions. Additionally, serum samples were also obtained. Moreover, 1 week before sacrifice, fasting blood was collected for glucose and insulin analysis to calculate the HOMA‐IR index using Matthews’ formula [23]. The detailed protocol is described in a previous publication [11].

### 2.5 Quantification of Circulating Glucose, Insulin, and Leptin Levels

Blood glucose was tested using an Accu‐Chek Glucometer (Roche Diagnostics, Barcelona, Spain). Enzyme‐linked immunosorbent assay kits were used for the quantification of insulin (Mercodia AB, Uppsala, Sweden) and leptin (R&D Systems, Minneapolis, MN, USA) in serum samples obtained in the fed state. Glucose and insulin levels were also measured in samples obtained in the fasted state (for HOMA‐IR index calculation).

### 2.6 Total RNA Isolation

Total RNA from hippocampus and PBMC was isolated using Tripure reagent (Roche Diagnostics Barcelona, Spain) following the manufacturer's protocol. RNA yield was quantified using a NanoDrop ND 1000 spectrophotometer (NanoDrop Technologies, Wilmington, DE, USA), and RNA integrity and purity were confirmed via 1% agarose gel electrophoresis.

### 2.7 Microarray Processing

Hippocampal RNA samples from NW (*n* = 8), MONW (*n* = 8), and MONW‐Lep (*n* = 9) animals were processed using Affymetrix GeneChip Rat Clariom S microarrays following standard protocols. RNA integrity and hybridization quality were confirmed before analysis. Normalization, background correction, and differential gene expression analysis were conducted using the *limma* [24] package in R version 4.0.4 and R Studio version 1.4.1717 software, and functional enrichment was performed with *ExpressAnalyst* and *Metascape*. Genes with a *p* value < 0.05 (*limma* *t*‐test) were considered significantly differentially expressed.This pipeline, including both sample processing and data analysis, followed the same methodology further described in [21] for PBMC transcriptomic profiling in the same animal cohort. Candidate gene validation (*Piwil1*) was performed by RT‐qPCR based on its relevance as a potential early biomarker of cognitive risk. Transcriptomic results obtained in the hippocampus were confronted with data from a microarray analysis performed in PBMC of the same groups: NW and MONW, as previously described [21].

### 2.8 RT‐qPCR Analysis

The mRNA expression of specific genes of interest was assessed by RT‐qPCR in both the hippocampus and PBMC. Fifty ng of RNA from each sample was reverse transcribed to cDNA using the iScript cDNA synthesis kit (BIO‐RAD, Madrid, Spain) in an Applied Biosystems 2720 Thermal Cycler. After cDNA synthesis, qPCR was performed to determine the mRNA expression of the analyzed genes. Each qPCR reaction utilized diluted (1/10 for hippocampus and 1/5 for PBMC) cDNA template, forward and reverse primers (10 µM), and Power Sybr Green PCR Master Mix (Applied Biosystems) under previously described reaction conditions [25]. The threshold cycle (Ct) was calculated using StepOne Software v2.0 (Applied Biosystems), and the relative mRNA expression was determined as a percentage of control (NW) rats using the 2^−ΔΔCt^ method [26]. Hippocampus and PBMC data were normalized against *Rplp0*, which has been previously validated as a useful constitutive gene [27]. Primers were obtained from Thermo Fisher Scientific (Life Technologies S.A, Madrid, Spain) and are described in the Supporting Information S1.

### 2.9 Statistical Analysis

Data are presented as means ± SEM. Statistical differences for anthropometric, circulatory, and cognitive parameters between the NW, MONW, and MONW‐Lep groups were assessed using Kruskal–Wallis test followed by Dunn post hoc method for pairwise comparisons. Differences in RT‐qPCR gene expression in hippocampus and PBMC samples were assessed using Mann–Whitney *U* test. Spearman's rank correlation coefficient (rho) was used to evaluate linear relationships between microarray gene expression data from the hippocampus with anthropometric, circulatory, and cognitive parameters. Statistical analyses were performed with RStudio, Build 351, R version 4.1.1, with significance defined at *p* < 0.05. For microarray data analysis, refer to section “2.7. Microarray Processing.”

## 3 Results

### 3.1 General Characteristics of Animal Models

As previously described [11] and summarized in Table 1, both MONW and MONW‐Lep groups showed increased adiposity compared to NW animals, despite similar body weight due to pair‐feeding with a HFD. However, only the MONW group exhibited features of metabolic dysfunction, including liver fat accumulation, elevated leptin levels, and signs of insulin resistance (elevated insulin and HOMA‐IR), which were not observed in MONW‐Lep rats, highlighting the protective effect of neonatal leptin treatment. Cognitive assessment revealed impaired working memory in the MONW group, while MONW‐Lep rats performed similarly to controls, supporting leptin's beneficial impact (as detailed in [6]).

**TABLE 1:** Most relevant anthropometric, circulatory, and cognitive parameters of the NW, MONW, and MONW‐Lep groups.

Anthropometric parameters	NW	MONW	MONW‐Lep
Body weight (g)	409 ± 13	416 ± 6	410 ± 6
Fat mass (%)	19.4 ± 0.50^a^	23.9 ± 1.8^b,^*	22.9 ± 1.2^a,b,^*
Visceral fat content (%)	5.99 ± 0.21^a^	7.79 ± 0.30^b,^*	7.31± 0.26^b,^*
Liver fat content (mg/g liver)	47.3 ± 3.9^a^	64.3 ± 4.2^b,^*	56.7 ± 5.2^a,b^

Circulatory parameters	NW	MONW	MONW‐Lep
Glucose‐fasting‐ (mg/dL)	101 ± 3^a^	120 ± 7^b,^*	114 ± 4^a,^*
Insulin‐fasting‐ (µg/L)	0.18 ± 0.02^a^	0.33 ± 0.05^b,^*	0.24 ± 0.03^a^
HOMA‐IR	1.06 ± 0.16^a^	2.45 ± 0.52^b,^*	1.7 ± 0.2^a^
Leptin‐feeding‐ (mg/mL)	12.8 ± 1.0^a^	18.1 ± 2.3^b,^*	12.5 ± 1.3^a^

Cognitive test	NW	MONW	MONW‐Lep
T‐Maze (% alternance)	100 ± 4^a^	76.8 ± 9.8 ^b,^*	87.0 ± 5.5^a,b^

*Note*: Data represent means ± SEM (*n* = 8 in the NW and MONW groups; *n* = 10 in the MONW‐Lep group). Statistical analysis: values not sharing a common letter (a, b) are significantly different (Kruskal–Wallis test followed by Dunn post hoc method for pairwise comparisons, *p* < 0.05); no letter indicates no significant differences. Additionally, asterisks (*) indicates a significant difference between MONW or MONW‐Lep versus NW (Mann–Whitney *U* test, *p* < 0.05).

### 3.2 Evaluation of Differentially Expressed Genes (DEGs) in the Hippocampus

**FIGURE 1:**
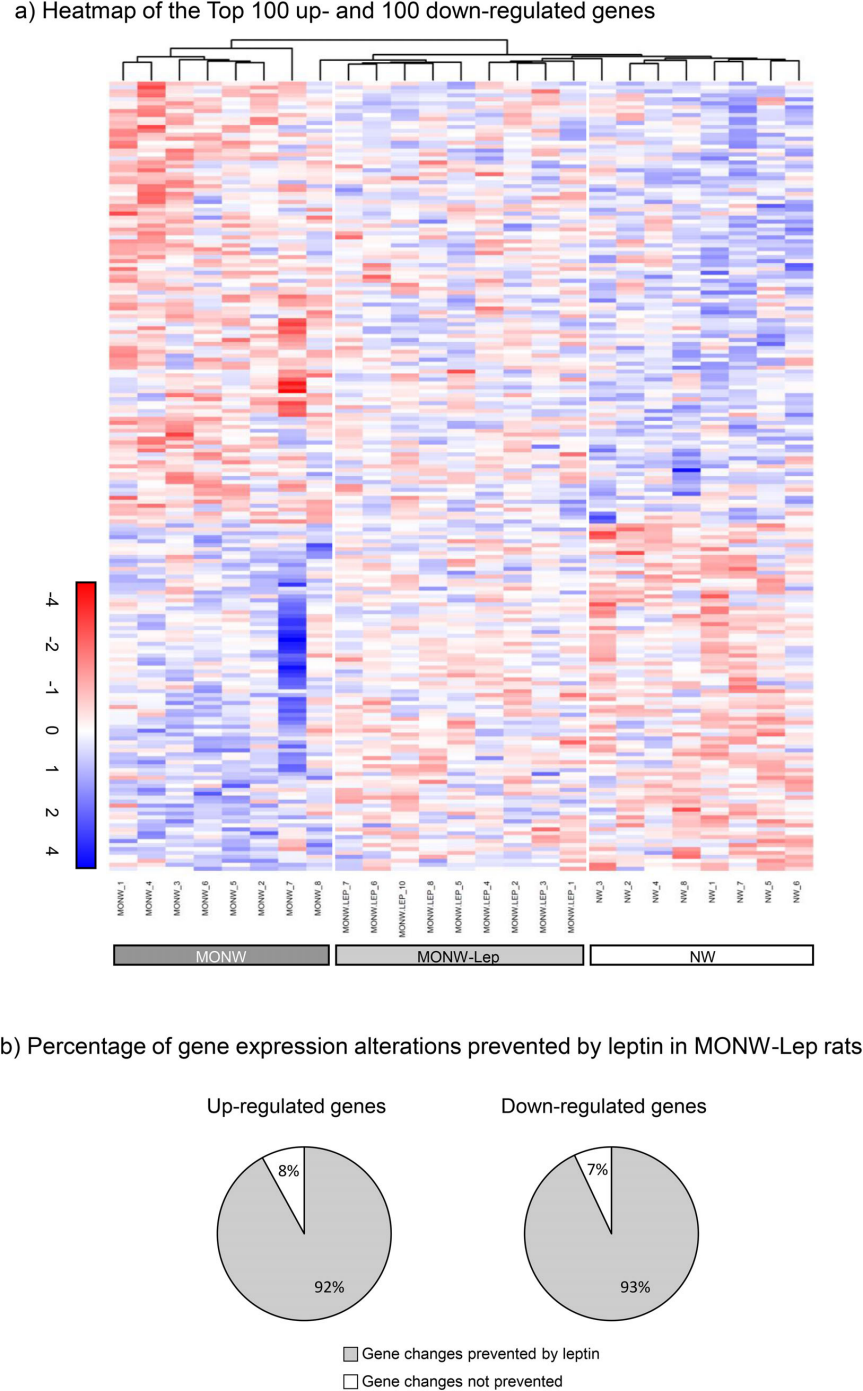
(a) Heatmap and hierarchical clustering representing the normalized gene expression patterns of the top 100 up‐ and 100 down‐regulated genes in the hippocampus from young MONW and NW rats. The heatmap illustrates both genes and samples clustered, with positive gene expression represented in blue and negative in red. (b) Percentage of gene expression alterations prevented by neonatal leptin supplementation in MONW‐Lep rats. The pie charts depict the proportion of up‐regulated (left) and down‐regulated (right) genes whose alterations were mitigated by leptin treatment compared to the genes altered in the MONW group. Heatmap was performed using RStudio.

**FIGURE 2:**
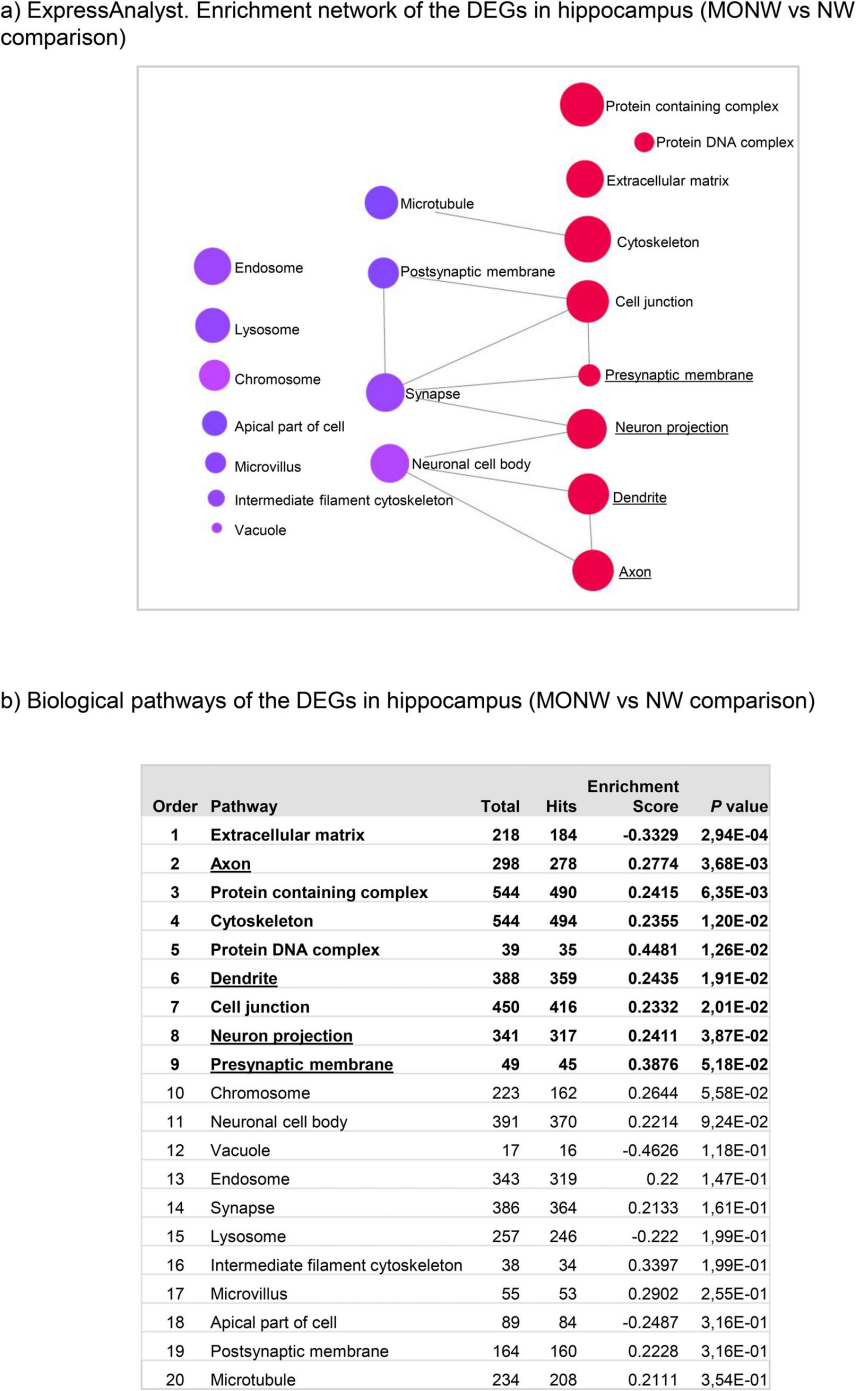
Enrichment network of all the microarray DEGs in the hippocampus of the MONW group in comparison to NW group using *ExpressAnalyst* from *NetworkAnalyst 3.0*. (a) Enrichment Network by the PANTHER database. Color intensity indicates the *p* value, with significant pathways shown in red. The size of the circle reflects the number of genes (Hits), while the lines connecting the nodes represent shared genes between the pathways. (b) Biological pathways of the DEGs. Data come from a Gene Set Enrichment Analysis using the PANTHER database. Pathways are ranked based on their *p* values. Pathways in bold have a *p* value < 0.05. Pathways underlined are associated with neurology.

**FIGURE 3:**
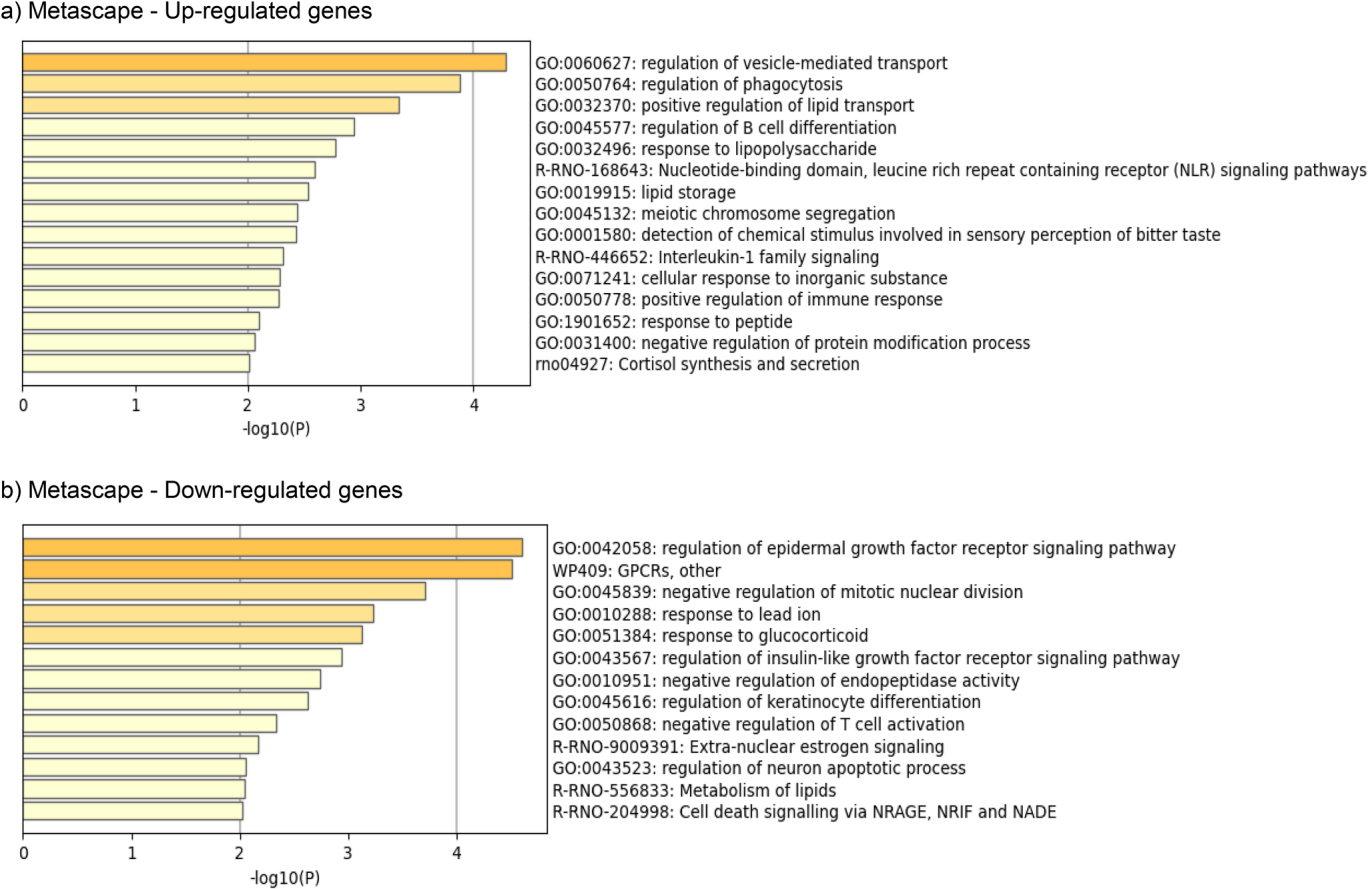
Functional enrichment analysis of DEGs in the hippocampus of the MONW group in comparison to the NW group using *Metascape*. Bar charts display the top enriched biological pathways identified by *Metascape* for (a) up‐regulated genes and (b) down‐regulated genes. The pathways are ranked by their significance (‐log10(*p* value)).

MONW phenotype induced by HF feeding in young animals had a relevant impact on the hippocampal gene expression pattern. This impact was less marked when animals received leptin during lactation. Hippocampal transcriptomic profiling revealed 304 DEGs in MONW versus NW and 234 DEGs in MONW‐Lep versus NW (*p* < 0.05). A heatmap analysis based on the top 100 up‐ and 100 down‐regulated DEGs allowed us to distinguish the three groups and showed that MONW‐Lep samples were more similar to NW than to MONW, suggesting mitigation of HFD‐induced transcriptomic changes by leptin (Figure 1a). Only 8% of MONW up‐regulated and 7% of down‐regulated genes remained altered in MONW‐Lep, further confirming this effect (Figure 1b). Enrichment Network (*ExpressAnalyst*) for all DEGs between the MONW and NW groups identified nine significantly altered pathways, including cognitive‐related terms (“Axon,” “Dendrite,” “Neuron projection,” “Presynaptic membrane”) (Figure 2). In contrast, only four pathways were altered in MONW‐Lep versus NW, reflecting a milder transcriptional disturbance (data not shown). Pathway analysis processed with *Metascape*, one focusing exclusively on up‐regulated genes and another on down‐regulated genes, identified processes related to inflammation and lipid metabolism (Figure 3).

### 3.3 Top 10 Up‐ and 10 Down‐Regulated Genes in the Hippocampus of MONW‐Like Animals

Among the 304 DEGs between MONW and NW animals, the top 10 up‐ and 10 down‐regulated genes (based on fold change) were prioritized. Interestingly, except for one gene (*Guca2a*, down‐regulated), the rest of these top 20 genes were not affected in the MONW‐Lep group, suggesting a protective effect of leptin. Each gene was manually categorized according to its associated biological process and general function, with a summary of previous research linking them to neurological functions or obesity (Supporting Information S2 and S3 for the up‐ and down‐regulated genes, respectively). Several top genes, including *Bhlhe23*, *Cpne5*, *Gopc*, *Rasgrp2*, and *Satb2* (up‐regulated) and *Alx1*, *Ccni*, *Prickle3*, *and Prmt7* (down‐regulated), are involved in neuronal development and function, and others, such as *Cpne5*, *Omd*, *Prokr2*, and *Stag3* (up‐regulated) have been previously associated with cognitive decline and AD. Moreover, *Mrap* (down‐regulated) has been related to obesity. Notably, *Cpne5* and *Olr1082* have been previously associated with both obesity‐related metabolic disorders and cognitive impairment, consistent with the MONW phenotype observed in these animals. The top‐regulated genes in MONW‐Lep animals included several with known links to improved cognition, further supporting the beneficial effect of leptin treatment (Table 2), as will be further elaborated in the discussion.

**TABLE 2:** Top 10 up‐ and 10 down‐regulated genes in the hippocampus of young 3.5‐month‐old metabolically obese, normal‐weight rats treated with leptin during lactation (MONW‐Lep) versus normal‐weight (NW) rats.

	Order	Gene symbol	Gene name	Sequence ID	Fold change	*p* value	Function
Up‐regulated	1	*Mylpf*	Myosin light chain, phosphorylatable, fast skeletal muscle	NM_012605.2	+1.34	0.002	Unknown function in the hippocampus
	2	*Olr664*	Olfactory receptor 664	NM_001000347.1	+1.29	0.001	Olfactory function
	3	*Olr363*	Olfactory receptor 363	NM_001000754.1	+1.29	0.004	Olfactory function
	4	*Cabyr*	Calcium binding tyrosine phosphorylation regulated	NM_001143893.2	+1.28	0.005	Calcium ion binding
	5	*Mcm3*	Minichromosome maintenance complex component 3	NM_001191805.2	+1.28	0.004	Cell cycle (replication)
	6	*Vegfd*	Vascular endothelial growth factor D	NM_031761.3	+1.26	0.023	Vascular angiogenesis
	7	*Tmem45b*	Transmembrane protein 45B	NM_001033067.2	+1.26	0.002	Immune response
	8	*Klrb1c*	Killer cell lectin like receptor B1C	NM_001085403.2	+1.26	0.007	Immune response
	9	*Tmem144*	Transmembrane protein 144	NM_001108551.1	+1.25	0.020	Carbohydrate transmembrane transporter activity
	10	*Ikzf3*	IKAROS family zinc finger 3	NM_001107047.1	+1.25	0.003	Immune response
Down‐regulated	1	*Slc5a1/Sglt1*	Solute carrier family 5 member 1	NM_013033.2	−1.39	0.003	Sodium‐glucose cotransport
	2	*Ctsw*	Cathepsin W	NM_001024242.1	−1.31	0.003	Immune response
	3	*Slc15a3*	Solute carrier family 15 member 3	NM_139341.1	−1.31	0.001	Amino acid transport
	4	*Chrna3*	Cholinergic receptor nicotinic alpha 3 subunit	NM_052805.3	−1.29	0.007	Synaptic transmission
	5	*Csap1*	Common salivary protein 1	NM_133622.2	−1.28	0.010	Codes for a salivary protein
	6	*Cdc6*	Cell division cycle 6	NM_001108298.1	−1.28	0.022	Cell cycle (replication)
	7	*Cysrt1*	Cysteine rich tail 1	NM_001109194.1t	−1.27	0.025	Unknown
	8	*Fmo3*	Flavin containing dimethylaniline monoxygenase 3	NM_053433.2	−1.27	0.009	Detoxification (cytochrome P450 pathway)
	9	*Khdc3*	KH domain containing 3, subcortical maternal complex member	NM_001106837.1	−1.27	0.011	Dendrite development
	10	*Olr141*	Olfactory receptor 141	NM_001001274.1	−1.26	0.001	Olfactory function

*Note*: Statistical analysis: limma moderated t‐statistic, *p* value < 0.05). Fold change (FC): MONW group/NW group, “+” indicates up‐regulation and, “−” indicates down‐regulation. Genes are ranked on FC.

### 3.4 Correlation Analysis of Microarray Data With Anthropometric and Circulatory Parameters

Gene expression levels of the top 10 up‐ and 10 down‐regulated hippocampal DEGs in MONW animals correlated with key metabolic traits, including fasting glucose, insulin, HOMA‐IR, and fat depots (Supporting Information S4a). Up‐regulated genes generally showed positive correlations, whereas down‐regulated ones were inversely correlated. These associations were largely absent in the MONW‐Lep group, suggesting that leptin treatment attenuated both gene expression changes and their link to metabolic alterations (Supporting Information S4b).

### 3.5 Validation of Cognitive Biomarkers in PBMC Through Microarray and RT‐qPCR Analyses

PBMC are known to reflect transcriptomic changes occurring in other tissues, including the brain [5, 6]. We compared hippocampal transcriptomic data with a previous microarray performed on PBMC from the same cohort of animals [21], aiming to identify non‐invasive early biomarkers of cognitive decline. Seven genes were found to be commonly regulated in both tissues, with changes in the same direction: two down‐regulated genes (*Olr729* and *Vom1r75*) and five up‐regulated genes (*Piwil1*, *RGD1559600*, *Spata3*, *Tas2r119*, and *Trib1*). Interestingly, three of these genes (*Olr729*, *Tas2r119*, and *Vom1r75*) are involved in sensory functions, which, when altered, are known to be linked to cognitive decline, AD, and obesity [28, 29, 30, 31]. Additionally, *Piwil1*, *Spata3*, and *Trib1* have previously been associated with obesity and its complications [32, 33, 34, 35]. Notably, *Piwil1* has also been linked to cognitive impairment and neurodegeneration, as it encodes a PIWI‐subfamily protein within the Argonaute family, involved in RNA silencing and neuronal function [36]. Specifically, *Piwil1* has been shown to inhibit axon regeneration in rat neurons, and its associated piRNAs have been found to be up‐regulated in brain samples from patients with AD, suggesting a role in neurodegenerative processes [36, 37, 38, 39]. Therefore, we considered *Piwil1* as a particularly relevant gene since it was associated with both metabolic and cognitive dysfunctions and presented altered expressions in both the hippocampus and PBMC of MONW‐like animals.

**FIGURE 4:**
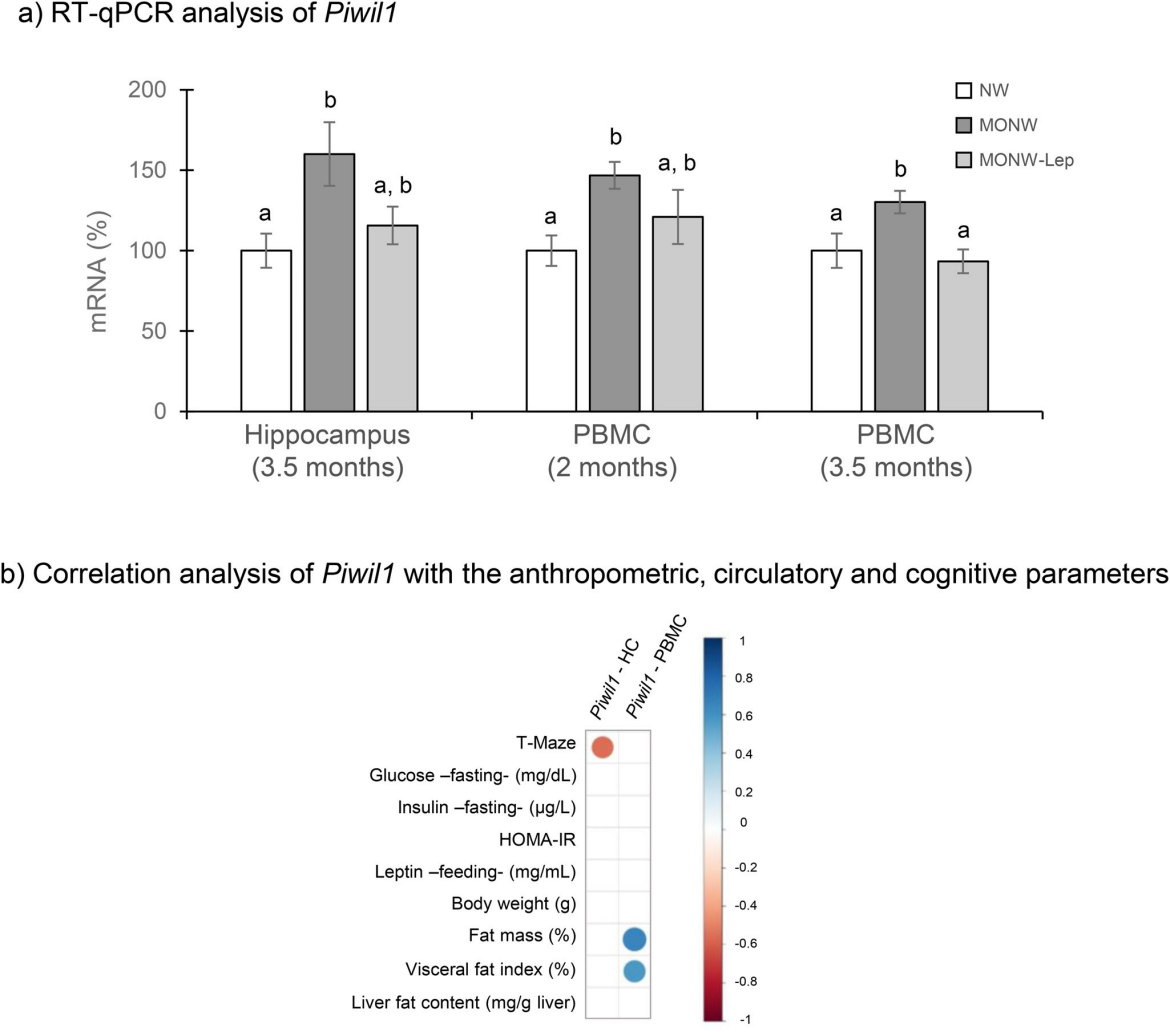
(a) *Piwil1* analyzed by RT‐qPCR in the hippocampus and PBMC of MONW, MONW‐Lep, and NW rats. Data represent means ± SEMs (*n* = 8–10) and are expressed as a percentage of the value of the NW group that was set to 100%. Statistics: values not sharing a common letter (a, b) are significantly different (Kruskal‐Wallis test followed by Dunn post hoc method for pairwise comparisons, *p* < 0.05); no letter indicates no significant differences. (b) Correlation analysis between hippocampal and PBMC gene expression of *Piwil1* (analyzed by RT‐qPCR) and anthropometric and circulatory parameters at 3.5 months of age. The panel shows correlations for MONW and NW animals. Spearman's rank correlation coefficient (rho) was calculated, and only significant correlations (*p* < 0.05) are shown as circles. Circle size and color represent the magnitude and direction of rho values, with positive correlations in blue and negative correlations in red. HC indicates hippocampus.

Thus, we selected *Piwil1* for further validation through RT‐qPCR in the hippocampus (3.5 months) and PBMC (2 and 3.5 months) from all experimental groups. This analysis confirmed its up‐regulation in MONW animals and showed that expression changes were already detectable in PBMC at 2 months. These alterations were mitigated by perinatal leptin supplementation (Figure 4a). Moreover, considering data of the NW and MONW animals, *Piwil1* expression in the hippocampus negatively correlated with T‐maze performance, and its expression in PBMC correlated positively with total and visceral fat content, supporting its potential as an early biomarker of MONW‐related cognitive dysfunction (Figure 4b).

## 4 Discussion

While the connection between obesity and dementia is well‐established [13], our group was the first in showing that the HFD‐induced MONW phenotype (characterized by increased adiposity and altered insulin sensitivity without overt obesity) can also lead to cognitive impairment both in adult and young rats [5, 6].These findings align with previous studies that highlight the vulnerability of the developing brain to early dietary insults, which can result in long‐term cognitive consequences [2]. Moreover, our previous research showed that PBMC can reflect gene expression changes occurring in the hippocampus, reinforcing their utility as accessible source of biomarkers of brain health [5, 6]. We also demonstrated that leptin supplementation during lactation prevents the cognitive deficits associated with HFD‐induced MONW, offering a potential early‐life intervention strategy [6].

Although our earlier studies focused on candidate genes, in this study, we expanded upon those findings by performing a comprehensive transcriptomic analysis of the hippocampus in young MONW‐like animals with cognitive impairment, as well as in animals that received leptin during lactation and preserved working memory. This allowed us to better characterize the molecular pathways affected by early high‐fat feeding and to investigate how leptin treatment may mitigate these changes.

### 4.1 Transcriptomic Alterations in the Hippocampus of MONW‐Like Animals

The hippocampus is especially sensitive to metabolic changes due to its role in energy balance and memory processes. In fact, our global transcriptomic analysis identified 304 DEGs between the MONW and NW groups, revealing notable alterations in pathways related to synaptic signaling. Pathway analysis of the top‐regulated genes with *Metascape* also highlighted the enrichment of lipid metabolism and inflammatory processes. The enrichment of lipid‐related pathways, including “Positive regulation of lipid transport” and “Lipid storage,” suggests altered lipid homeostasis in the hippocampus due to early HFD exposure. In addition, the up‐regulation of inflammation‐related pathways, such as “Regulation of B cell differentiation” and “Positive regulation of immune response,” points toward a pro‐inflammatory environment. These alterations may contribute to the cognitive deficits observed, as lipid imbalances and neuroinflammation are known to disrupt synaptic function in HFD‐induced obesity models [40]. Additionally, pathways like “Neuron projection” and “Presynaptic membrane” were significantly affected, suggesting impaired synaptic connectivity and plasticity. In this line, most of the top‐up‐regulated genes were involved in the “Regulation of vesicle‐mediated transport”, indicating a potential alteration in neurotransmitter release. The observed transcriptomic changes suggest that the cognitive impairments in the MONW group may be driven by a combination of lipid dysregulation, synaptic alterations, and inflammation.

Closer examination of the top 10 up‐ and 10 down‐regulated genes adds further insight. Many of the down‐regulated genes play critical roles in brain development. This is the case of *Alx1*, the most highly down‐regulated gene, which codes for a transcription factor involved in the development of forebrain mesenchyme in rodents [41]. Other highly down‐regulated genes, *Prmt7*, *Ccni*, and *Prickle3*, also code for proteins with roles involved in neuronal and brain development and function [42, 43, 44]. Therefore, their reduced expression may impact hippocampal structure and cognition. Also, in this line, we observed up‐regulation of *Bhlhe23*, encoding for transcriptional repressor of neuronal differentiation [45]. Other up‐regulated genes like *Gopc*, *Rasgrp2*, and *Satb2*, which support neuronal function, might represent compensatory responses to early‐life metabolic insults. The MONW group also showed down‐regulation of *Itprid1/Ccdc129*, a gene associated with neuroprotection in brain injury models [46], and whose reduction has been linked to microglial activation [47]. Its down‐regulation in our study could therefore be another indicator of neuroinflammation.

Disruption of energy metabolism is increasingly recognized as a contributor to cognitive dysfunction [48]. Proper glucose usage is critical for neuron function, and in fact, dysregulated glucose metabolism contributes to the onset and progression of cognitive impairment [49]. Interestingly, we observed down‐regulation of *Pklr*, encoding a key glycolytic enzyme, suggesting impaired glucose metabolism in the MONW hippocampus. Another down‐regulated gene, *Mrap*, plays a central role in glucocorticoid signaling through the melanocortin 2 receptor and thus, in energy regulation and body weight control [50]. Brain‐specific targeted deletion of *Mrap* homologs induces severe obesity in rodents [51] and its downregulation in adipose tissue of obese mice has been linked to impaired lipolysis and fat accumulation [52]. Given the similarities in gene expression between brain and adipose tissue [53], our results suggest that such adrenocorticotropic resistance may also occur in the hippocampus.

Obesity is considered a risk factor for dementia, sharing common disturbances such as inflammation [54]. Both pathologies also share sensory dysfunction, particularly olfactory deficits [29, 55]. In fact, impaired olfaction has been reported to predict cognitive decline among dementia‐free older adults [56]. Interestingly, we observed decreased expression of *Olr1082*, encoding for an olfactory receptor gene, in MONW‐like animals, which could be a biomarker of brain alteration related to both increased adiposity and dementia. Further connecting both conditions, we observed up‐regulation in *Cpne5* expression, previously associated with both AD and obesity [57], and *Prokr2*, a gene involved in circadian rhythms. *Prokr2* is up‐regulated in models of circadian disruption, which is itself implicated in cognitive decline [58]. Additional up‐regulated genes, such as *Stag3* and *Omd*, have also been linked to AD risk [59, 60]. For example, *Stag3*, which was the top‐upregulated gene in the hippocampus of our MONW‐like animals, has been identified as a susceptibility gene for AD in a genome‐wide association study in humans [59].

### 4.2 Protective Effects of Neonatal Leptin Treatment

We previously demonstrated that leptin intake during lactation prevents metabolic alterations in the MONW phenotype [11], including cognitive impairment [6]. As evidenced in the transcriptomic analysis in the hippocampus of the MONW‐Lep group, many of the alterations observed in the MONW group were mitigated. Specifically, leptin‐treated animals showed a hippocampal gene expression profile closely resembling that of the NW group, as confirmed by heatmap analysis. Only 8% of the up‐regulated and 7% of the down‐regulated DEGs found in MONW versus NW remained altered in MONW‐Lep animals. Among the top 20 regulated genes, only *Guca2a*, involved in signal transduction but with no known brain‐specific function, remained altered, clearly supporting the protective role of perinatal leptin intake.

Leptin's neuroprotective effects may derive from its established role in neurodevelopment, synaptic plasticity, and brain energy regulation [9, 12]. By maintaining synaptic integrity and reducing neuroinflammation, leptin appears to counteract the detrimental cognitive consequences of the MONW phenotype. Supporting this, several gene expression changes in the hippocampus of MONW‐Lep animals have previously been associated with cognitive improvement. For instance, *Slc5a1* (also known as *Sglt1*), the top down‐regulated gene in MONW‐Lep animals, encodes a sodium‐glucose cotransporter whose inhibition protects against cognitive impairment in mice [61, 62]. Additionally, *Vegf*, one of the top up‐regulated genes, encodes vascular endothelial growth factor (VEGF), whose hippocampal overexpression in adult rats increases neurogenesis and improves cognition [63]. These results suggest that leptin may exert its neuroprotective effects partly through modulation of glucose metabolism and vascular remodeling in the brain.

Improved cognitive performance in the MONW‐Lep group may also be linked to their better overall metabolic status. Leptin‐treated animals displayed healthier circulatory parameters, as previously described [11], and the correlations observed between top DEGs and metabolic variables in the MONW group disappeared in the MONW‐Lep group. Leptin treatment prevented hepatic fat accumulation and insulin resistance, enhanced insulin and leptin signaling capacity, reduced orexigenic gene expression in the hypothalamus, and promoted browning of retroperitoneal adipose tissue [11]. All these factors are neuroprotective, since increased adiposity, inflammation and insulin resistance are known to contribute to impaired cognition [13].

### 4.3 PBMC as Accessible Biomarkers for Cognitive Impairment

Early detection of cognitive disorders, including AD, is crucial for effective intervention. However, diagnoses are often made when the condition is already advanced. Given the challenges of obtaining brain samples, the search for accessible sources of blood biomarkers, such as PBMC, has become a priority in preventive medicine. In a previous study from our group [21], a PBMC microarray was performed in the same animal cohort. In that previous study, we reported that top‐regulated genes in PBMC of the MONW‐like animals were related to inflammation, pointing toward peripheral signatures potentially linked to neuroinflammatory responses in the brain.

In the present study, we compared the pathways identified in PBMC with those found in the hippocampus, using the same analytical tool (*ExpressAnalyst*), and found significant overlap. Specifically, “Cell junction,” “Cytoskeleton,” “Axon,” and “Extracellular matrix” pathways were disrupted in both tissues. These findings highlight the capacity of PBMC to reflect hippocampal alterations, underscoring their value as an early and accessible source of biomarkers of cognitive impairment caused by early‐life metabolic challenges, such as HFD intake.

Among the DEGs shared between hippocampus and PBMC, we identified *Trib1* (up‐regulated in the MONW‐like group), encoding a pseudokinase involved in the regulation of acute and chronic inflammation through modulation of inflammatory factors secretion, which is related to the occurrence of inflammation‐related pathologies [34]. However, *Piwil1*, overexpressed in both tissues, emerged as particularly relevant due to its dual association with obesity and cognitive function. This gene encodes a PIWI‐subfamily protein within the Argonaute family, characterized by conserved PAZ (Piwi/Argonaute/Zwille) and PIWI domains, involved in RNA silencing and stem cell self‐renewal [36]. In relation to cognition, PIWI proteins and its associated piRNAs have been shown to function in neurons [37]. A previous study demonstrated that *Piwil1* inhibits axon regeneration, and its knockdown promotes axonal regrowth in cultured adult rat sensory neurons [38]. Notably, the “Axon” pathway was among the commonly altered pathways in both tissues, reinforcing the relevance of *Piwil1*. Moreover, piRNAs linked to *PIWIL1* were found to be up‐regulated in brain samples of patients with AD, suggesting a potential role in neurodegeneration [39].

Beyond its role in neuronal function, *Piwil1* has been implicated in metabolic health. It was identified as a dynamical network biomarker in mice, predicting the onset of metabolic syndrome before clinical symptoms emerged [33]. This aligns with our observations in young MONW rats, which already showed early metabolic disturbances after just 11 weeks of HFD feeding, without overt obesity. Moreover, *Piwil1* was already up‐regulated in PBMC at 2 months of age (after only 5 weeks of HFD), highlighting its potential as a biomarker of early cognitive and metabolic risk. Additionally, the correlation between *Piwil1* expression, working memory performance, and key metabolic parameters, such as increased visceral fat, supports its candidacy as a biomarker for cognitive decline in the MONW phenotype. Notably, *Piwil1* expression remained unaltered in the MONW‐Lep group, both in PBMC and hippocampus, validating its usefulness as an early biomarker of metabolic and cognitive health/disease.

### 4.4 Conclusions and Perspectives

This study provides strong evidence that short‐term, isocaloric HFD exposure during early life induces hippocampal transcriptomic alterations in young MONW‐like rats, leading to cognitive deficits. Our findings offer mechanistic insights into how metabolic challenges during development can impact brain function. Notably, neonatal leptin supplementation during lactation protected against altered gene expression in the hippocampus due to HFD intake, preserving working memory. Additionally, we show that PBMC gene expression, particularly *Piwil1*, reflects hippocampal alterations and can serve as a non‐invasive early biomarker of cognitive health. All in all, these results highlight the critical role of early‐life nutrition for future cognitive function and support the use of transcriptomic biomarkers for early risk assessment and prevention. Considering the rise in chronic and neurodegenerative diseases, such as AD, and the lack of effective treatments, the emphasis should shift toward early and effective prevention strategies. Our findings contribute to the understanding of the relevance of promoting healthy nutritional habits from the earliest stages of life to mitigate long‐term cognitive risks. In this context, the identification of early biomarkers in accessible tissues such as PBMC will help identify at‐risk individuals to establish proper preventive strategies. Finally, it is important to remark that our study exclusively involved male animals to avoid hormonal fluctuations from the menstrual cycle in females. Future studies should explore potential sex differences.

## Conflicts of Interest

The authors declare no conflicts of interest.

## Supporting information




**Supporting File 1**: mnfr70262‐sup‐0001‐SuppMat.pdf.

**Supporting File 2**: mnfr70262‐sup‐0002‐SuppMat.pdf.

**Supporting File 3**: mnfr70262‐sup‐0003‐SuppMat.pdf.

**Supporting File 4**: mnfr70262‐sup‐0004‐SuppMat.pdf.

## Data Availability

The gene expression datasets generated during the current study are available in the Gene Expression Omnibus (GEO) repository, Series accession number “GSE264559.” The additional data generated in the study will be available from the corresponding author upon reasonable request. All authors confirm their willingness to share the IACUC‐approved protocol.
