# Supplementary material for: Altered Hippocampal Transcriptomic Profile Reveals Cognitive Impairment in Young Metabolically Obese, Normal‐Weight Rats, Prevented by Perinatal Leptin Intake
Source: Mol Nutr Food Res. 2025 Sep 13;69(22):e70262. doi: 10.1002/mnfr.70262 (PMC12643189; doi:10.1002/mnfr.70262)
Supplement: Supplementary file 1 — Supporting File 1: mnfr70262‐sup‐0001‐SuppMat.pdf. [file MNFR-69-e70262-s003.pdf]

Article title: Altered Hippocampal Transcriptomic Profile Reveals Cognitive Impairment in Young Metabolically Obese, Normal-Weight Rats, Prevented by Perinatal Leptin Intake

Authors' names: Carmen García-Ruano, Andrea Costa, Andreu Palou, Paula Oliver

Address and contact information of the corresponding author: Paula Oliver. Laboratory of Molecular Biology, Nutrition, and Biotechnology, Universitat de les Illes Balears. Cra. Valldemossa Km 7.5. E-07122-Palma, Mallorca, Spain. Phone: +34-971172548. E-mail: paula.oliver@uib.es

Supporting information 1: Nucleotide sequences of primers used for RT-qPCR amplification, and amplicon size.

Gene	Fordward Primer (5'-3')	Revese Primer (5'-3')	Amplicon Size (bp)
Piwil1	ACAACCCAAGACTGACCGTG	ACACTCCCACTTCTCACTGC	175
**Constitutive gene**			
Rplp0	GCAGCATCTACAGCCCAGAG	CCTTGACCTTTTCAGCCAGT	213
