# Supplementary material for: Altered Hippocampal Transcriptomic Profile Reveals Cognitive Impairment in Young Metabolically Obese, Normal‐Weight Rats, Prevented by Perinatal Leptin Intake
Source: Mol Nutr Food Res. 2025 Sep 13;69(22):e70262. doi: 10.1002/mnfr.70262 (PMC12643189; doi:10.1002/mnfr.70262)
Supplement: Supplementary file 2 — Supporting File 2: mnfr70262‐sup‐0002‐SuppMat.pdf. [file MNFR-69-e70262-s002.pdf]

Article title: Altered Hippocampal Transcriptomic Profile Reveals Cognitive Impairment in Young Metabolically Obese, Normal-Weight Rats, Prevented by Perinatal Leptin Intake

Authors' names: Carmen García-Ruano, Andrea Costa, Andreu Palou, Paula Oliver

Address and contact information of the corresponding author: Paula Oliver. Laboratory of Molecular Biology, Nutrition, and Biotechnology, Universitat de les Illes Balears. Cra. Valldemossa Km 7.5. E-07122-Palma, Mallorca, Spain. Phone: +34-971172548. E-mail: paula.oliver@uib.es

Supporting information 2: Top 10 up-regulated genes in hippocampus of young 3.5-month-old metabolically obese, normal-weight (MONW) vs normal-weight (NW) rats.

Order	Gene symbol	Gene name	Sequence ID	Fold change	*P* value	Biological process	Function	Description	DOI
1	*Stag3*	Stromal antigen 3	NM_053730.1	+1.42	0.021	Cell cycle	Cell division	Linked to AD susceptibility	DOI:10.1038/s41380-018-0112-7; DOI:10.1186/s13073-021-00959-y
2	*Upk1a*	Uroplakin 1A	NM_001108911.2	+1.36	0.017	Signal transduction	Transmembrane receptor	Involved in cell development, activation, growth and motility	
3	*Gopc*	Golgi associated PDZ and coiled-coil motif containing	NM_001393720.1	+1.36	0.018	Intracellular trafficking	Protein transport	Implicated in the targeting of subcellular sorting of neuronal proteins	DOI: 10.1007/s12035-021-02504-9
4	*Omd*	Osteomodulin	NM_031817.2	+1.34	0.018	Cell adhesion	Extracellular matrix remodeling	Plasma levels of the encoded protein are predictors of cognitive decline	DOI:10.1371/journal.pmed.1002931
5	*Prokr2*	Prokineticin receptor 2	NM_138978.2	+1.34	0.026	Signal transduction	G-protein-coupled receptor	Essential for the regulation of the circadian rhythm of behavior. Neuronal damage indicator (when up-regulated)	DOI:10.1021/acschemneuro.1c00238
6	*Rasgrp2*	RAS guanyl releasing protein 2	NM_001082977.2	+1.33	0.001	Signal transduction	Activation of GTPase activity	It contributes to signaling that regulates neuronal functions	DOI:10.1016/j.neuint.2022.105438
7	*Cpne5*	Copine 5	NM_001107616.2	+1.31	0.001	Signal transduction	Calcium-dependent lipid-binding protein	Suggested to play an important role in the development of the central nervous system. Genetic variants in the gene are related to AD and obesity	DOI:10.1016/j.brainres.2008.05.051 DOI:10.1016/j.jpsychires.2015.09.008
8	*Opn1sw*	Opsin 1, short wave sensitive	NM_031015.2	+1.30	0.008	Signal transduction	G-protein coupled receptor	Predicted to be involved in G-protein photoreceptor activity	DOI:10.1016/j.visres.2010.12.017
9	*Satb2*	SATB Homeobox 2	NM_001109306.1	+1.30	0.043	Gene expression regulation	Transcriptional factor	Regulation of cognition-related genes. Involved in long-term plasticity processes in the adult forebrain	DOI:10.7554/eLife.17361; DOI:10.15252/embj.2019103701
10	*Bhlhe23*	Basic helix-loop-helix family, member e23	NM_001109211.1	+1.29	0.006	Gene expression regulation	Transcriptional repressor	Repressor of neuronal differentiation	DOI:10.1016/j.neuron.2011.09.035

Top 10 up-regulated genes differentially expressed in the MONW vs the NW group (Limma moderated t-statistic, p-value <0.05). Fold change (FC): MONW group/NW group, "+" indicates up-regulation. Genes are ranked on FC. Abbreviation: AD for Alzheimer's disease.
