# Supplementary material for: Altered Hippocampal Transcriptomic Profile Reveals Cognitive Impairment in Young Metabolically Obese, Normal‐Weight Rats, Prevented by Perinatal Leptin Intake
Source: Mol Nutr Food Res. 2025 Sep 13;69(22):e70262. doi: 10.1002/mnfr.70262 (PMC12643189; doi:10.1002/mnfr.70262)
Supplement: Supplementary file 3 — Supporting File 3: mnfr70262‐sup‐0003‐SuppMat.pdf. [file MNFR-69-e70262-s001.pdf]

Article title: Altered Hippocampal Transcriptomic Profile Reveals Cognitive Impairment in Young Metabolically Obese, Normal-Weight Rats, Prevented by Perinatal Leptin Intake

Authors' names: Carmen García-Ruano, Andrea Costa, Andreu Palou, Paula Oliver

Address and contact information of the corresponding author: Paula Oliver. Laboratory of Molecular Biology, Nutrition, and Biotechnology, Universitat de les Illes Balears. Cra. Valldemossa Km 7.5. E-07122-Palma, Mallorca, Spain. Phone: +34-971172548. E-mail: paula.oliver@uib.es

Supporting information 3: Top 10 down-regulated genes in the hippocampus of young 3.5-month-old metabolically obese, normal-weight (MONW) vs normal-weight (NW) rats.

Order	Gene symbol	Gene name	Sequence ID	Fold change	*P* value	Biological process	Function	Description	DOI
1	*Alx1*	ALX homeobox 1	NM_012921.1	-1.45	0.044	Gene expression regulation	Transcriptional factor	Development of forebrain mesenchyme in rodents	DOI: 10.3389/fcell.2022.777887
2	*Olr1082*	Olfactory receptor 1082	NM_173335.2	-1.41	0.038	Signal transduction	G-protein-coupled receptor	Involved in sensory perception of smell. Alteration of olfactory receptor's expression is linked to AD and obesity	DOI:10.2147/NDT.S104886; DOI: 10.3390/metabo14010016
3	*Mrap*	Melanocortin 2 receptor accessory protein	NM_001135834.1	-1.39	0.000	Signal transduction	Melanocortin receptor	Involved in energy and body weight control. Its deletion in the brain is related to severe obesity in rodents	DOI:10.1126/science.1233000
4	*Prmt7*	Protein arginine methyltransferase 7	NM_001014153.1	-1.38	0.017	Gene expression regulation	Methylation	Involved in stem cells and neuronal development. Its deficiency is linked to impairment of social behaviors in humans	DOI:10.1155/2021/6241600; DOI:10.1038/s12276-020-0417-x
5	*Pklr*	Pyruvate kinase L/R	NM_012624.3	-1.35	0.009	Glucose metabolism	Glycolysis	Codes for a key regulatory enzyme in the glycolytic pathways. Decreased glycolytic capacity is linked to AD	DOI:10.3389/fncel.2021.774569; DOI:10.3389/fnins.2020.530219
6	*Ccni*	Cyclin I	NM_001105998.1	-1.35	0.041	Cell cycle	Cyclin-dependent kinase regulation	Codes for an activator of CDK5, playing a key role in brain development and cognition	DOI:10.1242/jcs.147553;
7	*Elapor2*	Endosome-lysosome associated apoptosis and autophagy regulator family member 2	NM_001109345.1	-1.35	0.009	Apoptosis	Lysosome-mediated autophagy	Regulator of apoptosis and autophagy through the lysosomal pathway, relevant processes for brain health	DOI:10.3389/fncel.2020.00039
8	*Guca2a*	Guanylate cyclase activator 2A	NM_013118.1	-1.30	0.022	Signal transduction	cGMP signaling	Positive regulation of guanylate cyclase activity. No function described in the brain	
9	*Itprid1/ Ccdc129*	ITPR interacting domain containing 1	NM_001191973.1	-1.28	0.002	Signal transduction		Predicted to enable signaling receptor binding activity. Downregulated in a model of activated microglia	DOI: 10.3390/cells12182213
10	*Prickle3*	Prickle planar cell polarity protein 3	NM_001014110.1	-1.27	0.002	Signal transduction	Planar cell polarity pathway	Involved in regulation of cellular polarity and neural tube formation	DOI:10.7554/eLife.37881

Top 10 down-regulated genes differentially expressed in the MONW vs the NW group (Limma moderated t-statistic, p-value <0.05). Fold change (FC): MONW group/NW group, "-" indicates down-regulation. Genes are ranked on FC. Abbreviation: AD for Alzheimer's disease.
