# Supplementary material for: Altered Hippocampal Transcriptomic Profile Reveals Cognitive Impairment in Young Metabolically Obese, Normal‐Weight Rats, Prevented by Perinatal Leptin Intake
Source: Mol Nutr Food Res. 2025 Sep 13;69(22):e70262. doi: 10.1002/mnfr.70262 (PMC12643189; doi:10.1002/mnfr.70262)
Supplement: Supplementary file 4 — Supporting File 4: mnfr70262‐sup‐0004‐SuppMat.pdf. [file MNFR-69-e70262-s004.pdf]

Article title: Altered Hippocampal Transcriptomic Profile Reveals Cognitive Impairment in Young Metabolically Obese, Normal-Weight Rats, Prevented by Perinatal Leptin Intake

Authors' names: Carmen García-Ruano, Andrea Costa, Andreu Palou, Paula Oliver

Address and contact information of the corresponding author: Paula Oliver. Laboratory of Molecular Biology, Nutrition, and Biotechnology, Universitat de les Illes Balears. Cra. Valldemossa Km 7.5. E-07122-Palma, Mallorca, Spain. Phone: +34-971172548. E-mail: paula.oliver@uib.es

Supporting information 4:

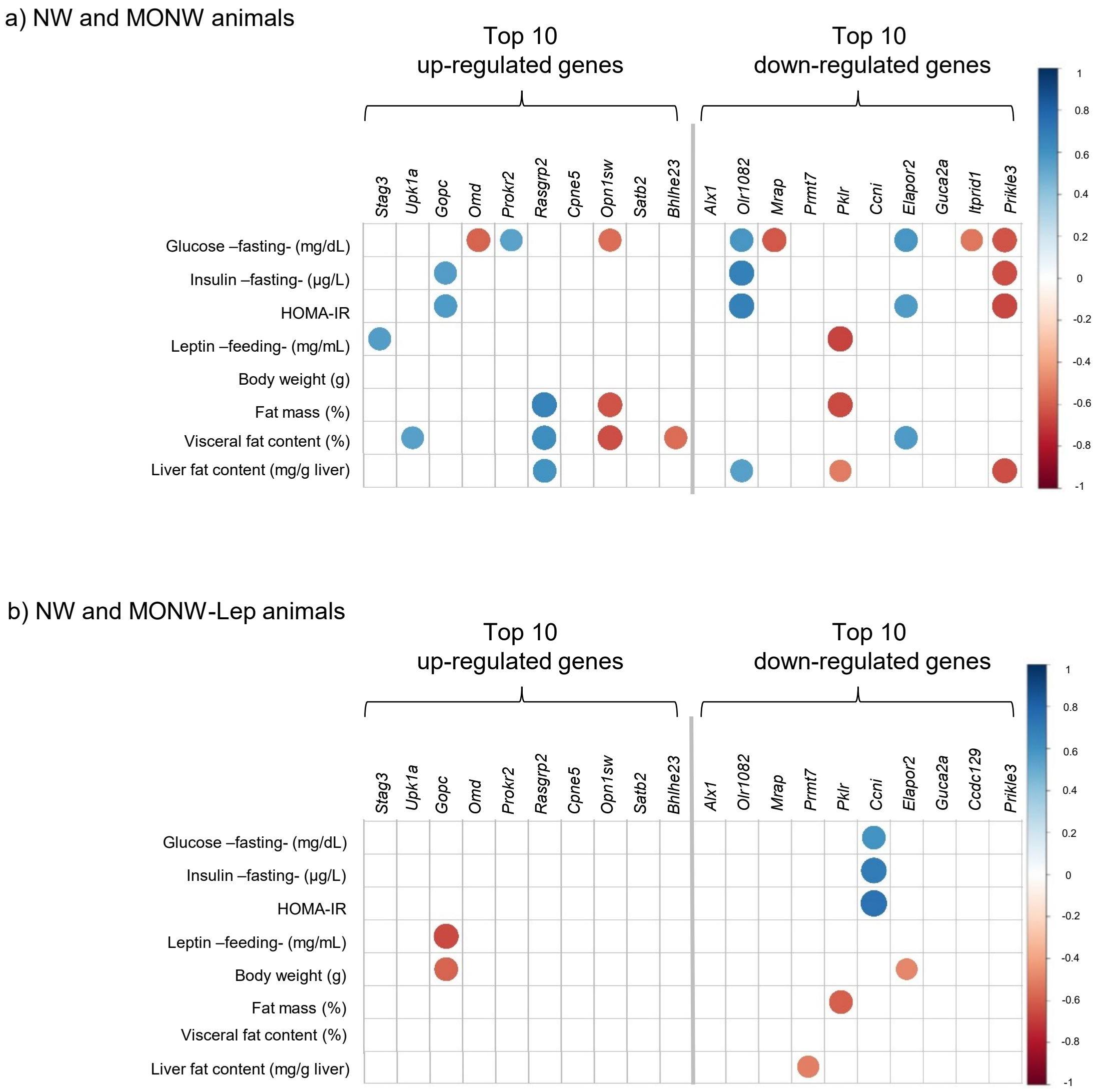

Correlation analysis between hippocampal gene expression of the top 10 up- and 10 down-regulated genes and anthropometric and circulatory parameters at 3.5 months of age. Panels show correlations for (A) young metabolically obese, normal-weight (MONW) and normal-weight (NW) animals, and (B) young metabolically obese, normal-weight with leptin supplementation (MONW-Lep) and normal-weight (NW) animals. Spearman's rank correlation coefficient (rho) was calculated, and only significant correlations ($p < 0.05$) are shown as circles. Circle size and color represent the magnitude and direction of rho values, with positive correlations in blue and negative correlations in red.
